# Neonicotinoid insecticides hinder the pupation and metamorphosis into adults in a crabronid wasp

**DOI:** 10.1038/s41598-020-63958-w

**Published:** 2020-04-27

**Authors:** Petr Heneberg, Petr Bogusch, Alena Astapenková, Milan Řezáč

**Affiliations:** 1 Charles University, Third Faculty of Medicine, Prague, Czech Republic; 2 University of Hradec Králové, Faculty of Science, Hradec Králové, Czech Republic; 3 Crop Research Institute, Functional Biodiversity Group, Prague, Czech Republic

**Keywords:** Zoology, Environmental sciences

## Abstract

Neonicotinoid insecticides are associated with a decline in the diversity and distribution of bees and wasps (Hymenoptera: Aculeata). The effects of neonicotinoids on the metamorphosis of aculeates have never been addressed in detail; however, recent evidence suggests that neonicotinoids induce wing abnormalities. We hypothesized that the metamorphosis success of bees and wasps differs in response to contact exposure to field-realistic concentrations of neonicotinoid insecticides or in response to combined exposure to neonicotinoid insecticides and benzimidazole fungicides. We treated prepupae of the model crabronid wasp *Pemphredon fabricii* with field-realistic concentrations of four neonicotinoids, acetamiprid, imidacloprid, thiacloprid and thiamethoxam, and/or with the benzimidazole fungicide thiabendazole. Treatment with acetamiprid or imidacloprid decreased the pupation rates to only 39% and 32%, respectively. Treatment with thiacloprid or thiamethoxam did not affect the pupation rate when applied alone, but the subsequent treatment of thiacloprid- or thiamethoxam-treated prepupae with thiabendazole led to significant decreases in pupation rates. A high concentration of acetamiprid, which severely affected the pupation rates, had moderate effects on metamorphosis into adults, resulting in 53% metamorphosis success (as opposed to 95% metamorphosis success in the water-treated group). However, imidacloprid or thiamethoxam treatment resulted in only 5%-10% metamorphosis success into adults. Overall survival decreased in response to treatment with any of the neonicotinoids or benzimidazoles or their combinations, with extremely low survival (<2%) following combined treatment with imidacloprid and thiabendazole or thiamethoxam and thiabendazole. In conclusion, neonicotinoids alter insect metamorphosis success, which can be further potentiated by their combination with other agrochemicals, such as benzimidazoles.

## Introduction

Neonicotinoid insecticides are well known for their association with a decline in bee and wasp diversity and distribution^1–5^. Although the molecular basics of the mechanism of action of neonicotinoids are known^6–9^, the effects of neonicotinoids on the metamorphosis of aculeate hymenopterans (bees and wasps) have never been addressed in detail. The important exception is the recent study by Friedli *et al*.^10^, who found that neonicotinoids cause an increased frequency of wing abnormalities and fluctuating wing asymmetry in haploid neonicotinoid-exposed *Apis mellifera* drones when compared to diploid workers. As wing veins develop during insect metamorphosis, this observation should be considered to be indirect evidence of the effects of neonicotinoids on the metamorphosis of aculeate hymenopterans. Increased neonicotinoid-induced mortality also coincided with metamorphosis timing in *A. mellifera*^11^. These observations are further supported by similar effects reported in other insect taxa^12,13^. Complete insect metamorphosis includes the formation of the intermediate nonfeeding pupal (chrysalis) stage, which allows the formation of adult structures from pupal tissues or from the imaginal discs that have been present since embryogenesis^14^. Insect metamorphosis is tightly regulated by the endocrine system, a metabolic switch to the Warburg cycle^15^, the preferential utilization of carbohydrates and lipids instead of amino acids^16,17^, extensive tissue remodeling and transdetermination^18^. Concerning the effects of anthropogenic stressors, little information is available. Detoxifying mechanisms are likely restrained during pupation, as defecation, rejection of cells from the alimentary canal surface and exuviae shedding cannot be performed. Delayed post-exposure effects related to metamorphosis have been reported; these effects consisted of the survival of toxin-exposed larvae until but not beyond metamorphosis^19–23^.

Instead of the exposure to neonicotinoids alone, the insects face the mixtures of agrochemicals. The exposure to neonicotinoids often occurs together with the exposure to azole fungicides (benzimidazoles, triazoles and imidazoles), which are broadly sprayed in foliar applications as antifungal agents^24,25^. In the past, azoles were also used directly in apiculture as acaricides to treat *Varroa* infections (cymiazole)^26^ and proposed to be used as fungicides to treat *Ascosphaera aphis* infections causing chalkbrood (propiconazole)^27^. In 2010s, synergism between azole fungicides and neonicotinoid insecticides was reported from several hymenopteran species^28–34^; only a decade-older paper by Bayer-based Schmuck *et al*.^35^ has reported the absence of effects. These putative synergistic effects could explain in part the repeated observations of beekeepers, who report aberrancies in brood rearing following the use of both neonicotinoids and benzimidazoles^27,36^. In the present study, we tested thiabendazole, a benzimidazole fungicide carrying a 1,3-thiazol-4-yl substituent at position 2. Thiabendazole is an azole fungicide that was previously found to have multiplicative effects on insect metamorphosis when applied to prepupae that were previously exposed to agrochemicals during their development, although it did not induce premature death like another closely related benzimidazole, flusilazole^28^. Thiabendazole is used in both agriculture and human/veterinary medicine.

To test the effects of neonicotinoids on the metamorphosis of bees and wasps (Hymenoptera: Aculeata), we employed the model crabronid wasp *Pemphredon fabricii*. This species survives well under laboratory conditions, and its collection is not associated with any conservation issues, as it is available in large quantities. It is the most abundant aculeate hymenopteran in common reed beds, where it uses old galls induced by frit flies *Lipara* spp. for nesting^37^. As with other crabronids, *P. fabricii* is predatory and feeds its larvae with aphids^38^. The development of *P. fabricii* includes five larval instars; the development itself takes only one to three weeks, after which the larva stops feeding, defecates and sheds to develop into a prepupa. The prepupae of the second generation emerge in late summer and are present in the reed galls until the next spring and metamorphose in April or later, depending on the ambient temperature^38^. The metamorphosis of *P. fabricii* was reported to be delayed in prepupae that were previously collected from areas with high concentrations of heavy metals and in those that were collected from field margins and later experimentally treated with azole fungicides^28^.

We hypothesized that the metamorphosis success of the model crabronid wasp, *P. fabricii*, differs in response to contact treatment with field-realistic concentrations of neonicotinoid insecticides. We further hypothesized that these effects are strengthened by combined exposure to both neonicotinoids and benzimidazole fungicides and/or in individuals that developed in anthropogenic environments that are strongly contaminated with heavy metals.

## Materials and methods

We collected cigar galls that were induced at least a year ago by *Lipara lucens* at terrestrial stands of common reed *Phragmites australis*. We longitudinally cut these galls and collected prepupae of *P*. *fabricii*, which were abundantly present in these galls. We sampled the galls in two distinct habitat types; the sampled habitats were never subjected to any application of benzimidazole or neonicotinoid insecticides and differed in origin. We compared natural, large, well-preserved terrestrial reed beds near centuries-old fish ponds (Dlouhopolský fish pond near Dlouhopolsko, 50.17 N, 15.31E, 1504 prepupae; Proudnický fish pond near Hradištko II, 50.12 N, 15.40E, 577 prepupae) with an anthropogenic habitat with high heavy metal concentrations^39^ represented by a tailing pond used for the waste from uranium processing (Bytíz, 49.69 N, 14.06E, 433 prepupae). We collected the galls on March 24, 2019 and kept them at 4 °C until excision. The prepupae were already subjected to diapause, which resulted in the synchronization of their developmental stage regardless of their exact age. The collection of *P. fabricii* prepupae only after diapause also substantially increases the rearing success^28^. Prepupae do not take any food after their diapause period; therefore, only contact application of the tested compounds was possible.

We excised the prepupae and placed them in 96-well plates (Brand, Wertheim, Germany), individually placing one prepupa in each well. We continued to keep the prepupae at 4 °C and ≥90% humidity. Just before the start of the experiment on April 4, 2019 (day 0), we checked the examined materials for pupation and excluded any individuals that proceeded spontaneously to pupation from the experiment. On day 0, we applied the field-realistic concentrations of neonicotinoid insecticides as described below. We applied four neonicotinoid insecticide formulations, each in a dilution recommended by their manufacturers for use in spraying crops to eliminate pest insects. The tested insecticides consisted of the neonicotinoids acetamiprid (formulated as Mospilan 20 SP; 126.0 ng cm^−2^ or 512.4 ng cm^−2^), imidacloprid (Confidor 200 OD; treatment 591.75 ng cm^−2^ or 1183.5 ng cm^−2^), thiacloprid (Biscaya 240 OD; treatment 472.7 ng cm^−2^ or 704.3 ng cm^−2^) and thiamethoxam (Actara 25 WG; treatment 178.5 ng cm^−2^ or 210.0 ng cm^−2^)^40^. We excluded some other neonicotinoid insecticides, such as clothianidin, as these are used only for the treatment of seeds before they are sown. We applied the tested compounds to the whole surface of the tested 96-well plates, which contained the immobile prepupae, by spraying them with a controlled amount of the neonicotinoids in distilled water or distilled water alone using the Potter Precision Laboratory Spray Tower (Burkard Scientific, Uxbridge, UK).

On day 1, 24 h following the application of neonicotinoids, we checked the experimental individuals for any pupation events. Following the check, we applied the benzimidazole fungicide thiabendazole (Sigma-Aldrich, St. Louis, MO, cat. No. T5535; 100% purity). The recommended field use rate of thiabendazole is 16,000 mg L^−1^, 220–780 kg ha^−1^ for foliar application to protect *T. aestivum* and 75–10,000 mg L^−1^, 420–1000 g ha^−1^ for foliar application to protect apples *Malus pumila* and pears *Pyrus communis*^41^. We applied thiabendazole at 2.5 μg cm^−2^ (the norm allows the application of up to, e.g., 7.8 μg cm^−2^ in Spain) to the whole surface of the tested 96-well plates, which contained the immobile prepupae, by spraying them with a controlled amount of the thiabendazole in distilled water or distilled water alone using the Potter Precision Laboratory Spray Tower (Burkard Scientific, Uxbridge, UK). Therefore, the tested concentration of thiabendazole was below the maximum recommended concentration for its foliar use.

After the administration, we allowed the droplets to dry for 24 h at 23 °C, and then we placed the plates with prepupae into an atmosphere with >95% humidity and continued to keep them at 23 °C until the end of the experiments^28^. We monitored the experimental individuals daily for 14 days and then performed a final check on day 19. We recorded the time to molting, time to full development of wings and mortality.

Data are shown as the frequencies within the treated groups. We used the χ^2^ test with the Yates correction for continuity to analyze the differences in binary values. We further applied the Bonferroni correction for repeated tests to avoid false-positive test outcomes. We applied one-way ANOVA to analyze the differences in the timing of the observed effects. The groups of prepupae that were treated with neonicotinoids and the control (water-treated) group included from 177 to 192 individuals. The groups of prepupae that were subjected to combined treatments with neonicotinoids and thiabendazole, and the control group (treated with thiabendazole only), included 88 to 96 individuals. The analyses were conducted in SigmaPlot 12.0. Raw data are provided in Table S1.

## Results

### Effects of sampling sites

**Figure 1:**
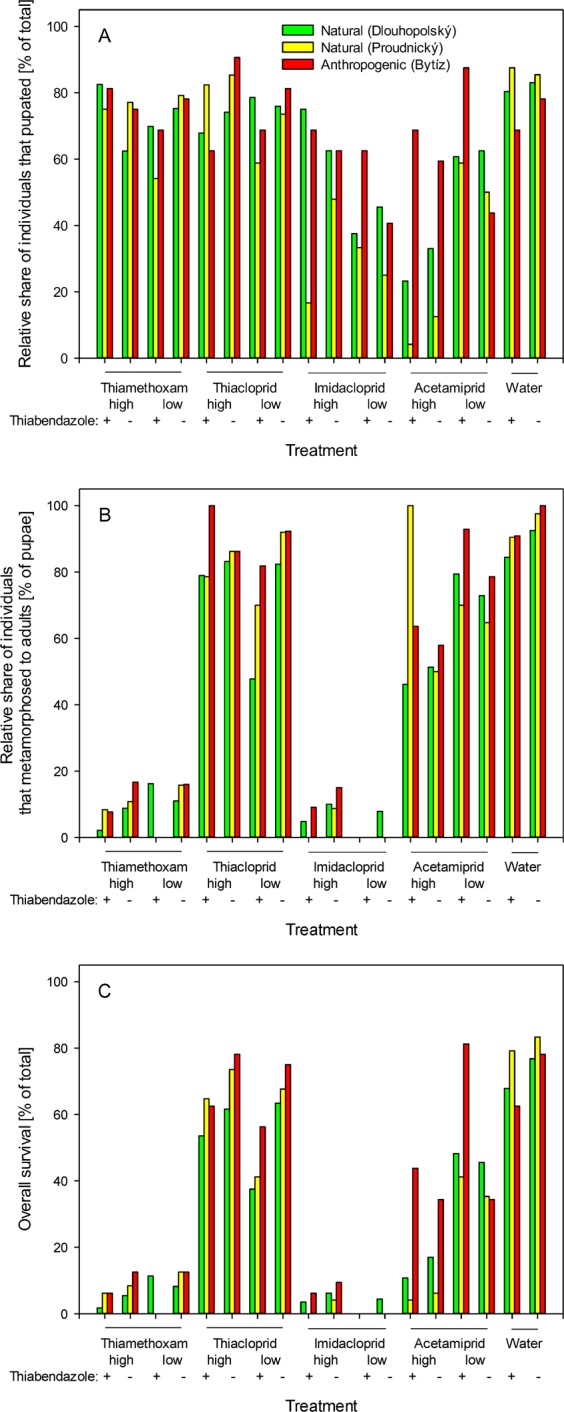
The effects of topical application of neonicotinoid insecticides combined or not with exposure to a benzimidazole fungicide on the success of *Pemphredon fabricii* metamorphosis. (**A**) Relative share of individuals that pupated [% of total]. (**B**) Relative share of individuals that metamorphosed from pupae to adults [% of pupae]. (**C**) Overall survival [% of total]. The data were analyzed separately for individuals collected from two sampling sites in natural habitats and one sampling site in an anthropogenic habitat. The raw data are provided in Table S1.

The water-treated prepupae (n = 192) had 82.8% pupation success, and 95.0% of those that pupated managed to complete metamorphosis into adults. Pupation success did not differ between the water-treated prepupae that were collected at the natural and anthropogenic sites (χ^2^ test with Yates and Bonferroni corrections at n = 18, *p* > 0.05 each). However, pupation success differed between the prepupae that were collected at the natural and anthropogenic sites when treated with low thiacloprid, high or low acetamiprid, thiabendazole and the combinations of high or low acetamiprid with thiabendazole (χ^2^ test with Yates and Bonferroni corrections at n = 18, *p* < 0.05 each). In treatments with acetamiprid (both with and without thiabendazole), the prepupae that originated from natural habitats displayed more than twice lower pupation rates (26.9% *vs*. 59.4% for high acetamiprid, and 17.5% *vs*. 68.8% for high acetamiprid in combination with thiabendazole when comparing the prepupae from natural and contaminated habitats, respectively). However, we observed the opposite direction of differences following the treatments with low acetamiprid or thiamethoxam (Fig. 1a).

At the end of the experiment (day 19), all the individuals that were included in the experiment, regardless of their treatment, either proceeded to metamorphose into adults or died. The successful metamorphosis into the adult stage did not differ between the prepupae that were collected at the natural and anthropogenic sites for water-treated as well as for neonicotinoid- and benzimidazole-treated prepupae (χ^2^ test with Yates and Bonferroni corrections at n = 18, *p* > 0.05 each). The only exceptions consisted of high or low acetamiprid combined with thiabendazole (χ^2^ test with Yates and Bonferroni corrections at n = 18, *p* < 0.01 each); in these cases, less individuals metamorphosed into adults among those that were collected from natural habitats (Fig. 1a).

Concerning the number of deaths, there was no difference between the prepupae that were collected at the natural and anthropogenic sites for water-treated as well as for most of the groups of neonicotinoid- and benzimidazole-treated prepupae (χ^2^ test with Yates and Bonferroni corrections at n = 18 *p* > 0.05 each). However, in groups that were treated with high thiamethoxam, high thiacloprid, low thiacloprid in combination with thiabendazole, high acetamiprid or high/low acetamiprid in combination with thiabendazole, more deaths were experienced among prepupae that were collected in natural habitats (χ^2^ test with Yates and Bonferroni corrections at n = 18 *p* < 0.05 each). The observation of sampling site-specific differences requires more attention in further studies as the effect of contaminants or other factors cannot be excluded. The obtained data were evaluated together in all of the further tests.

### Pupation rates

The observed effects can be split into two categories – the binary responses and the time elapsed to the respective response. Concerning the binary data, we focused on the relative numbers of individuals that completed pupation, the relative numbers of individuals that metamorphosed into adults and overall survival during the course of the experiment. The relative number of individuals that pupated differed from that observed in the water-treated cohort when treated with any of the tested concentrations of imidacloprid or acetamiprid and when treated with the higher concentration of thiamethoxam (χ^2^ test with Yates and Bonferroni corrections at n = 17, *p* < 0.01 each). Changes in the absolute values of pupation rates were most prominent when high concentrations of imidacloprid or acetamiprid were applied; these conditions decreased the pupation rates to only 39.6% and 32.3%, respectively. In contrast, the treatment with thiacloprid at any of the two tested concentrations and the treatment with the lower concentration of thiamethoxam did not lead to significant changes in pupation rates compared to those observed in the water-treated prepupae (χ^2^ test with Yates and Bonferroni corrections at n = 17, *p* > 0.05 each). The treatment of prepupae with thiabendazole alone did not alter the pupation rates. However, the combined treatment with neonicotinoids and thiabendazole led to significant decreases in pupation rates in all experimental settings. The combined treatment caused significant decrease in pupation rates when combined with any of the formulations of neonicotinoids except of high thiamethoxam, independently on whether the particular formulation of the neonicotinoid alone was insufficient to observe an effect. Therefore, the treatment with high or low thiacloprid concentrations or the low thiamethoxam concentrations negatively affected the pupation rate when combined with the thiabendazole treatment (χ^2^ test with Yates and Bonferroni corrections at n = 17, *p* < 0.01 each), although none of these treatments alone had any effect (Fig. 1A; Table 1).

**Table 1:** Overall effects of topical application of neonicotinoid insecticides combined or not with exposure to a benzimidazole fungicide thiabendazole on the success of *Pemphredon fabricii* metamorphosis.

1^st^ + 2^nd^ treatment	Relative share of individuals that pupated [% of total]	Relative share of individuals that metamorphosed from pupae to adults [% of pupae]	Relative share of individuals that metamorphosed from pupae to adults [% of total]	N
Thiamethoxam high + Thiabendazole	80.9	4.2*	3.4*	89
Thiamethoxam high + H_2_O	68.3*	10.9*	7.4*	189
Thiamethoxam low + Thiabendazole	63.6*	1.8*	1.1*	88
Thiamethoxam low + H_2_O	76.1	10.0*	7.6*	184
Thiacloprid high + Thiabendazole	68.9*	82.3	56.7*	90
Thiacloprid high + H_2_O	79.2	84.4	66.9*	178
Thiacloprid low + Thiabendazole	73.0*	56.9*	41.6*	89
Thiacloprid low + H_2_O	76.3	86.7	66.1*	177
Imidacloprid half + Thiabendazole	59.4*	5.3*	3.1*	96
Imidacloprid half + H_2_O	58.9*	10.6*	6.3*	192
Imidacloprid high + Thiabendazole	40.6*	0.0*	0.0*	96
Imidacloprid high + H_2_O	39.6*	5.3*	2.1*	192
Acetamiprid high + Thiabendazole	26.0*	56.0*	14.6*	96
Acetamiprid high + H_2_O	32.3*	53.2*	17.2*	192
Acetamiprid low + Thiabendazole	65.2*	81.0	52.8*	89
Acetamiprid low + H_2_O	56.7*	72.3*	41.0*	178
H_2_O + Thiabendazole	80.2	87.0	69.8*	96
H_2_O + H_2_O	82.8	95.0	78.6	192

*χ^2^ test with Yates and Bonferroni corrections *vs*. the H_2_O + H_2_O treatment at n = 18 *p* < 0.01. Number of individuals treated in each group is indicated.

to supplementary tables.

### Success of metamorphosis to adults

The relative number of individuals that metamorphosed from pupae into adults differed between the water-treated cohort and those treated with any of the tested concentrations of thiamethoxam or imidacloprid (χ^2^ test with Yates and Bonferroni corrections at n = 17, *p* < 0.01 each). Interestingly, the high concentrations of acetamiprid, which severely affected the pupation rates, had only a moderate effect on the relative share of individuals that metamorphosed into adults, although the differences from that observed in the water-treated controls were still significant (χ^2^ test with Yates and Bonferroni corrections at n = 17 *p* < 0.01 each). Treatment with imidacloprid resulted in metamorphosis into adult success rates of only 5.3% and 10.6% when considering the treatment with the higher and lower imidacloprid concentrations, respectively. Treatment with thiamethoxam resulted in metamorphosis into adult success rates of only 10.9% and 10.1% when considering the treatment with the higher and lower thiamethoxam concentrations, respectively. In contrast, treatment with the high acetamiprid concentrations resulted in a metamorphosis success rate of 53.2%. Thiabendazole alone did not have a statistically significant effect on the success of metamorphosis into adults. However, in combination with low concentrations of thiacloprid, it led to a significant decrease in the rate of successful metamorphosis into adults of 86.7% to 56.9% (χ^2^ test with Yates and Bonferroni corrections at n = 17, *p* < 0.01), despite any of these treatments alone having no effect (Fig. 1B; Table 1).

### Overall survival

There were no individuals that survived over the whole period of the experiment and did not metamorphose into the adults; therefore, the numbers of surviving individuals were equal to the numbers of individuals that managed to metamorphose from prepupae into pupae and subsequently into adults. Concerning survival, all tested groups differed significantly from the water-treated control (χ^2^ test with Yates and Bonferroni corrections at n = 17, *p* < 0.01 each). We observed the lowest survival rates in individuals that were subjected to the combined treatment with either high imidacloprid and thiabendazole (no surviving individuals) or low thiamethoxam and thiabendazole (a single surviving individual). Generally, all modes of imidacloprid and thiamethoxam treatments led to a survival rate below 10%. The combination of imidacloprid or thiamethoxam with thiabendazole further decreased the survival values to less than half of the values that were obtained with imidacloprid or thiamethoxam alone. Interestingly, we did not observe this multiplicative effect in acetamiprid-treated individuals. When the water-treated and thiacloprid-treated individuals were subjected to thiabendazole treatment, they displayed only a small decrease in their survival rates, which reached approximately 10% under all of these conditions (Fig. 1C).

### Timing of observed effects

**Figure 2:**
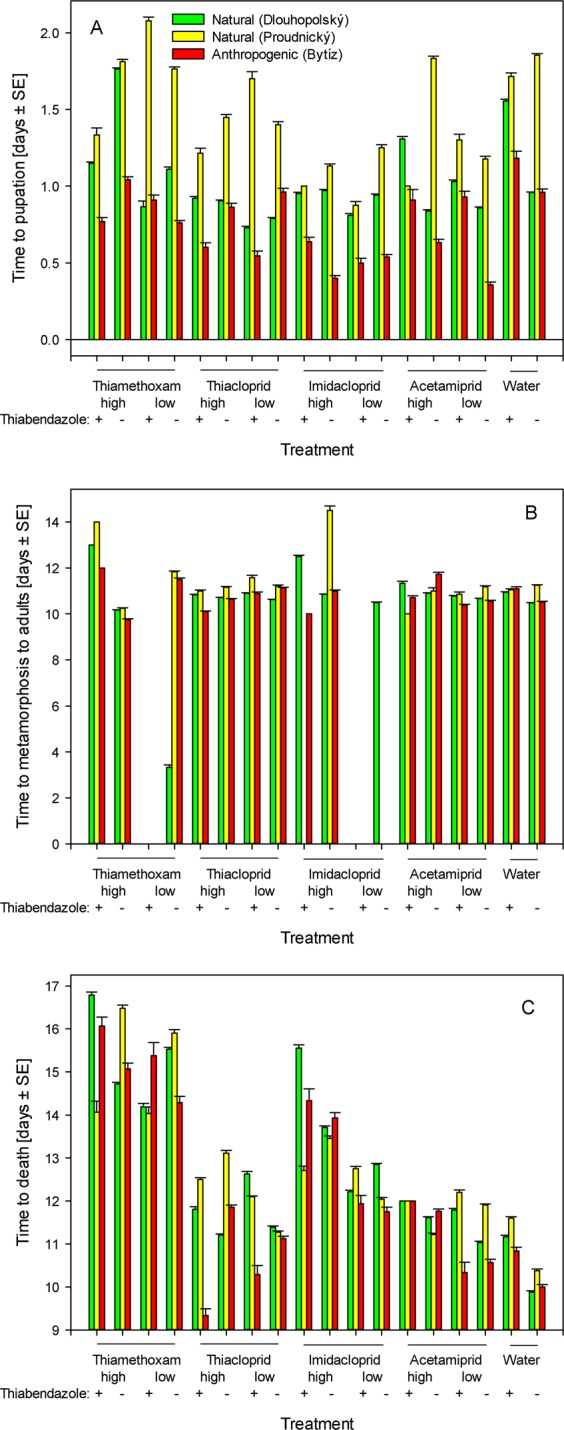
The effects of topical application of neonicotinoid insecticides combined or not with exposure to a benzimidazole fungicide on the timing of *Pemphredon fabricii* metamorphosis and delayed death. (**A**) Time to pupation, expressed as the number of days that elapsed from treatment to pupation ± SE. (**B**) Time to metamorphosis into adults, expressed as the number of days that elapsed from treatment to metamorphosis into adults ± SE. (**C**) Time to death, expressed as the number of days that elapsed from treatment to death ± SE. The data were analyzed separately for individuals collected from two sampling sites in natural habitats and one sampling site in an anthropogenic habitat. The raw data are provided in Table S1.

Because the tested individuals were collected in early spring, the time to pupation was very short and the mean time to pupation lasted between one and two days in all tested groups (Fig. 2A). The mean time from experiment onset to metamorphosis into adults lasted between 10 and 11 days for most of the tested groups. There were some exceptions associated with treatments that were generally associated with very low survival. In these cases (high imidacloprid and high thiamethoxam combined with thiabendazole), the time to metamorphosis into adults was prolonged to 13 or 14 days (Fig. 2B). The third observed variable, the time to death, was inversely related to the death rates and was the shortest in the water-treated group. In comparison to that observed in the water-treated group, the time to death was longer in all neonicotinoid-treated and thiabendazole-treated groups (one-way ANOVA, *p* < 0.01). We observed the longest times to death in thiamethoxam-treated individuals (Fig. 2C), which had difficulties with metamorphosis into adults but not pupation (Fig. 1).

## Discussion

In the present study, we provide the first conclusive data regarding the effects of a broad spectrum of neonicotinoid insecticides on the success of pupation and metamorphosis into adults of aculeate hymenopterans. We further provide evidence that the effects of another class of agrochemicals, benzimidazoles, synergize with the effects of neonicotinoids. Although the effects of benzimidazoles on insect metamorphosis were rather mild, which is consistent with previous observations^28^, we found that the effects of the administration of neonicotinoids and benzimidazoles had more prominent effects than treatment with any of these compounds alone.

Although we provide the first direct evidence of the effects of neonicotinoids on insect metamorphosis success, information on several of the same indices was already available. Shortly before this paper was submitted, Friedli *et al*.^10^ addressed the effects of neonicotinoids on the development of wings, and found that neonicotinoids altered wing venation. Another study published several years ago showed that neonicotinoids increase the fluctuating asymmetry of insect wing size^42^. The presence of wing abnormalities is often used to measure developmental precision in insects^43^, and wing asymmetry is often associated with the presence of external stressors, including exposure to toxic compounds or pathogens^44,45^. Aberrant wing development is a typical result of altered insect metamorphosis because this process is characteristic of the final molt from pupa to the adult individual in holometabolan species^46^.

Another piece of evidence showing the putative effects of neonicotinoids on insect metamorphosis stems from reports that found imidacloprid to affect juvenile hormone levels in various nontarget insects^47–51^. The mechanism of the effects of neonicotinoids on juvenile hormone levels is poorly understood. One link could consist of vitellogenin, the mRNA of which is increased upon neonicotinoid exposure in *A. mellifera*^52^. An increase in vitellogenin is likely to be associated with a decrease in juvenile hormone levels^53,54^. At this point, the effects of neonicotinoids could be potentiated by benzimidazoles, which are known to alter the levels of other key regulators of insect metamorphosis, Krüppel homolog 1^55^ and ecdysone^56^ or steroid hormone biosynthesis in general^57^. Benzimidazoles also alter the action of microtubules, which are necessary for, e.g., tracheole migration and evagination^58^ and wing disc evagination^59^.

The present study suggests that the effects of particular neonicotinoid insecticides differ from one another. Moreover, some of the treatments had more prominent effects on only metamorphosis into adults, whereas other treatments affected both pupation and metamorphosis into adults. In general, the tested treatments were associated with delayed post-exposure effects. All tested treatments were associated with increased mortality. However, this mortality did not manifest at the same time as the naturally present mortality observed in the water-treated group. Instead, all these groups displayed a seemingly prolonged time to death (Fig. 2C). The individuals that died first prolonged the period during which they were in the prepupal or pupal stage or underwent incomplete metamorphosis. They remained in these stages much longer than the water-treated individuals and longer than the individuals that were treated identically but managed to complete metamorphosis. In most cases, death occurred only after this period of excessively long presence in the prepupal or pupal stage and was likely a result of unsuccessful metamorphosis events. The observed effects should not be considered sublethal; these instead represent delayed lethal effects. Therefore, the outcomes of the present study support recent calls for the prolonged monitoring of experimental organisms, e.g., *A. mellifera* larvae, as delayed toxicity may start to manifest at day 11^60^, at day 19^61^, etc. In contrast, regulatory guidelines suggest that studies of acute toxicity should last for up to four days only and that studies of chronic toxicity should take only up to ten days when examining caged adult *A. mellifera*^62^.

The differential effects of neonicotinoids could stem from differences in their binding sites. In the American cockroach, *Periplaneta americana*, imidacloprid acts on antagonist of nicotinic receptor 1 (nAChR1), whereas it does not act on nAChR2^63–66^. In contrast, acetamiprid binds to nAChR2^67,68^. However, acetamiprid is rapidly biotransformed to a number of compounds, including 6-chloronicotinic acid, which remains stable in insect bodies except gut-free abdomen for at least 72 hours, which likely explains the toxicity of acetamiprid^69^. Actually, the 6-chloronicotinic acid is a metabolite that is common to all chloropyridinyl neonicotinoids, and is therefore formed from imidacloprid and thiacloprid as well^70,71^. The mechanism of action of thiacloprid is less understood. Similarly to other neonicotinoid, its main metabolization pathway includes N-demethylation; five different sites of attack are known but reports on the activity of its metabolites are lacking^72^. Thiamethoxam, which belongs to a second generation of neonicotinoids, acts only poorly on nAChRs^65,73,74^, and binds mixed nicotinic/muscarinic receptors^75^, causing strong depolarization of cercal afferent/giant interneuron synapses^76^. However, the thiamethoxam metabolite clothianidin acts as nAChr1 and nAChR2 agonist^73,76,77^; also metabolic N-desmethylation of thiamethoxam increases its affinity to nAChRs^73,78,79^. Many assays revealed differences in the toxicity of individual neonicotinoids. For example, Iwasa *et al*.^80^ examined acute toxicity of neonicotinoids to *A. mellifera* and reported LD_50_ for imidacloprid of only 18 ng per bee, whereas LD_50_ for thiacloprid was higher by three orders of magnitude, at 14.6 μg per bee, which is a striking difference given that the recommended application rates of these insecticides are roughly the same.

In conclusion, we identified previously unreported decreases in pupation success and metamorphosis into adult success in response to treatment with neonicotinoids, which need to be taken in account when performing risk assessment of neonicotinoids on bees and wasps^81^. The individuals that did not manage to pupate or metamorphose were alive for several additional days and then died. The effects of neonicotinoids were strengthened by the combined exposure to both neonicotinoids and benzimidazole fungicide. In contrast, there were no additive effects of prior exposure of the tested prepupae to other anthropogenic stressors, which were mimicked by collecting some of the tested individuals from a tailing pond used for the waste from uranium processing that was strongly contaminated with heavy metals. Further research should address whether similar effects can be observed in commercially exploited species of aculeate hymenopterans and in other insect taxa.

## Supplementary information

Supplementary information.

## References

[1] Whitehorn PR, O’Connor S, Wackers FL, Goulson D (2012). Neonicotinoid pesticide reduces bumble bee colony growth and queen production. Science.

[2] Dicks L (2013). Bees, lies and evidence-based policy. Nature.

[3] Rundlöf M (2015). Seed coating with a neonicotinoid insecticide negatively affects wild bees. Nature.

[4] Tsvetkov N (2017). Chronic exposure to neonicotinoids reduces honey bee health near corn crops. Science.

[5] Woodcock BA (2017). Country-specific effects of neonicotinoid pesticides on honey bees and wild bees. Science.

[6] Casida JE (2011). Neonicotinoid metabolism: compounds, substituents, pathways, enzymes, organisms, and relevance. J. Agric. Food Chem..

[7] Rand EED (2015). Detoxification mechanisms of honey bees (Apis mellifera) resulting in tolerance of dietary nicotine. Sci. Rep..

[8] Ihara M, Matsuda K (2018). Neonicotinoids: molecular mechanisms of action, insights into resistance and impact on pollinators. Curr. Opin. Insect Sci..

[9] Wang X (2018). Mechanism of neonicotinoid toxicity: impact on oxidative stress and metabolism. Annu. Rev. Pharmacol. Toxicol..

[10] Friedli A, Williams GR, Bruckner S, Neumann P, Straub L (2020). The weakest link: Haploid honey bees are more susceptible to neonicotinoid insecticides. Chemosphere.

[11] Tavares DA (2017). Exposure of larvae to thiamethoxam affects the survival and physiology of the honey bee at post-embryonic stages. Environ. Pollut..

[12] Bao H (2009). Sublethal effects of four insecticides on the reproduction and wing formation of brown planthopper, Nilaparvata lugens. Pest Manag. Sci..

[13] Zhang J, Yuan F, Liu J, Chen H, Zhang R (2010). Sublethal effects of nitenpyram on life-table parameters and wing formation of Nipalarvata lugens (Stål) (Homoptera: Delphacidae). Appl. Entomol. Zool..

[14] Gilbert, L. & Frieden, E. Metamorphosis: a problem in developmental biology. Plenum Press, New York and London (2013).

[15] Tennessen JM, Baker KD, Lam G, Evans J, Thummel CS (2011). The Drosophila estrogen-related receptor directs a metabolic switch that supports developmental growth. Cell Metab..

[16] Chikaraishi Y, Ogawa NO, Doi H, Ohkouchi N (2011). 15N/14N ratios of amino acids as a tool for studying terrestrial food webs: a case study of terrestrial insects (bees, wasps, and hornets). Ecol. Res..

[17] Judd TM, Fasnacht MP (2017). A nutritional profile of the trap-nesting wasp Trypoxylon lactitarse (Hymenoptera: Crabronidae): comparison of sexes and overwintering and non-overwintering generations. Insects.

[18] Beira JV, Paro R (2016). The legacy of Drosophila imaginal discs. Chromosoma.

[19] Büyükgüzel K, Yazgan Ş (2002). Effects of antimicrobial agents on the survival and development of larvae of Pimpla turionellae L. (Hymenoptera: Ichneumonidae) reared on an artificial diet. Turk. J. Zool..

[20] Bots J, De Bruyn L, Snijkers T, Van den Branden B, Van Gossum H (2010). Exposure to perfluorooctane sulfonic acid (PFOS) adversely affects the life-cycle of the damsefly Enallagma cyathigerum. Environ. Pollut..

[21] Wesner JS, Kraus JM, Schmidt TS, Walters DM, Clements WH (2014). Metamorphosis enhances the effects of metal exposure on the mayfly, Centroptilum triangulifer. Environ. Sci. Technol..

[22] Dinh KV (2016). Delayed effects of chlorpyrifos across metamorphosis on dispersal-related traits in a poleward moving damselfly. Environ. Pollut..

[23] Debecker S, Dinh KV, Stoks R (2017). Strong delayed interactive effects of metal exposure and warming: latitude-dependent synergisms persist across metamorphosis. Environ. Sci. Technol..

[24] Singh S (2016). Toxicity, monitoring and biodegradation of the fungicide carbendazim. Environ. Chem. Lett..

[25] David A (2016). Widespread contamination of wildflower and bee-collected pollen with complex mixtures of neonicotinoids and fungicides commonly applied to crops. Environ. Int..

[26] Cabras P, Martini MG, Floris I, Spanedda L (1994). Residues of cymiazole in honey and honey bees. J. Apicult. Res..

[27] Huntzinger CI, James RR, Bosch J, Kemp WP (2008). Laboratory bioassays to evaluate fungicides for chalkbrood control in larvae of the alfalfa leafcutting bee (Hymenoptera: Megachilidae). J. Econ. Entomol..

[28] Heneberg P, Bogusch P, Astapenková A (2019). The effects of contact exposure to azole fungicides on insect metamorphosis. Crop Protect..

[29] Thompson HM, Fryday SL, Harkin S, Milner S (2014). Potential impacts of synergism in honeybees (Apis mellifera) of exposure to neonicotinoids and sprayed fungicides in crops. Apidologie.

[30] Zhu YC, Yao J, Adamczyk J, Luttrell R (2017). Synergistic toxicity and physiological impact of imidacloprid alone and binary mixtures with seven representative pesticides on honey bee (Apis mellifera). PLoS ONE.

[31] Sgolastra F (2017). Synergistic mortality between a neonicotinoid insecticide and an ergosterol-biosynthesis-inhibiting fungicide in three bee species. Pest Manag. Sci..

[32] Raimets R (2018). Synergistic interactions between a variety of insecticides and an ergosterol biosynthesis inhibitor fungicide in dietary exposures of bumble bees (Bombus terrestris L.). Pest Manag. Sci..

[33] Sgolastra F (2018). Combined exposure to sublethal concentrations of an insecticide and a fungicide affect feeding, ovary development and longevity in a solitary bee. Proc. Biol. Sci..

[34] Willow J, Silva A, Veromann E, Smagghe G (2019). Acute effect of low-dose thiacloprid exposure synergized by tebuconazole in a parasitoid wasp. PLoS ONE.

[35] Schmuck R, Stadler T, Schmidt HW (2003). Field relevance of a synergistic effect observed in the laboratory between an EBI fungicide and a chloronicotinyl insecticide in the honeybee (Apis mellifera L, Hymenoptera). Pest Manag. Sci..

[36] Mussen EC, Lopez JE, Peng CYS (2004). Effects of selected fungicides on growth and development of larval honey bees, Apis mellifera L. (Hymenoptera: Apidae). Environ. Entomol..

[37] Heneberg P, Bogusch P, Astapenková A (2014). Reed galls serve as an underestimated but critically important resource for an assemblage of aculeate hymenopterans. Biol. Conserv..

[38] Bogusch P, Havelka J, Astapenková A, Heneberg P (2018). New type of progressive provisioning as a characteristic parental behaviour of the crabronid wasp Pemphredon fabricii (Hymenoptera Crabronidae). Ethol. Ecol. Evol..

[39] Fernandes M, Franklin MR, Veiga LHS, Freitas P, Gomiero LA (1996). Management of uranium mill tailing: Geochemical processes and radiological risk assessment. J. Environ. Radioact..

[40] Řezáč M, Řezáčová V, Heneberg P (2019). Contact application of neonicotinoids suppresses the predation rate in different densities of prey and induced paralysis of common farmland spiders. Sci. Rep..

[41] FAO. THIABENDAZOLE (65). Available from,http://www.fao.org/fileadmin/templates/agphome/documents/Pests_Pesticides/JMPR/Evaluation97/Thiaben.PDF. Cited as November 24, 2019 (1997).

[42] Rosa AS (2016). Consumption of the neonicotinoid thiamethoxam during the larval stage affects the survival and development of the stingless bee, Scaptotrigona aff. depilis. Apidologie.

[43] Lopuch S, Tofilski A (2016). The relationship between asymmetry, size and unusual venation in honey bees (Apis mellifera). Bull. Entomol. Res..

[44] Chang X, Zhai B, Wang M, Wang B (2007). Relationship between exposure to an insecticide and fluctuating asymmetry in a damselfly (Odonata, Coenagriidae). Hydrobiologia.

[45] Gerard M (2018). Stressful conditions reveal decrease in size, modification of shape but relatively stable asymmetry in bumblebee wings. Sci. Rep..

[46] Belles X (2019). The innovation of the final moult and the origin of insect metamorphosis. Philos. Trans. R. Soc. Lond. B Biol. Sci..

[47] Sclar DC, Gerace D, Cranshaw WS (1998). Observation of population increases and injury by spider mites (Acari: Tetranychidae) on ornamental plants treated with imidacloprid. J. Econ. Entomol..

[48] James DG, Price TS (2002). Fecundity in twospotted spider mite (Acari: Tetranychidae) is increased by direct and systemic exposure to imidacloprid. J. Econ. Entomol..

[49] Wang AH (2005). Selective insecticide-induced stimulation on fecundity and biochemical changes in Tryporyza incertulas (Lepidoptera: Pyralidae). J. Econ. Entomol..

[50] Yu YS, Xue S, Wu JC, Wang F, Yang GQ (2007). Changes in levels of juvenile hormone and molting hormone in larvae and adult females of Chilo suppressalis (Lepidoptera: Pyralidae) after imidacloprid applications to rice. J. Econ. Entomol..

[51] Yu Y, Shen G, Zhu H, Lu Y (2010). Imidacloprid-induced hormesis on the fecundity and juvenile hormone levels of the green peach aphid Myzus persicae (Sulzer). Pestic. Biochem. Physiol..

[52] Christen V, Mittner F, Fent K (2016). Molecular effects of neonicotinoids in honey bees (Apis mellifera). Environ. Sci. Technol..

[53] Amdam GV, Omholt SW (2002). The regulatory anatomy of honeybee lifespan. J. Theor. Biol..

[54] Amdam GV, Omholt SW (2003). The hive bee to forager transition inhoneybee colonies: the double repressor hypothesis. J. Theor. Biol..

[55] Wang K (2018). Transcriptome analysis of newly emerged honeybees exposure to sublethal cerbendazim during larval stage. Front. Genet..

[56] Ashburner M (1972). Ecdysone induction of puffing in polytene chromosomes of Drosophila melanogaster: Effects of inhibitors of RNA synthesis. Exp. Cell Res..

[57] Rybczynski, R. The prothoracicotropic hormone. In: Gilbert, L. L., Iatrou, K. & Gill, S. (Eds.) Comprehensive molecular insect science. Elsevier, Oxford, pp. 61–123 (2005).

[58] Hasskari E, Oberlander H, Stephens RE (1973). Microtubules and tracheole migration in wing disks of Galleria mellonela. Dev. Biol..

[59] Quan G-X, Kanke E, Kawasaki H (1998). Isolation and particular expression of a new β-tubulin gene in wing discs during metamorphosis of Bombyx mori. J. Seric. Sci. Jpn..

[60] Rondeau, G. *et al*. Delayed and time-cumulative toxicity of imidacloprid in bees ants and termites. *Sci. Rep.* **4**, 5566 (2014). 10.1038/srep05566 PMC4081892 24993452

[61] Dechaume-Moncharmont F-X, Decourtye A, Hennequet-Hantier C, Pons O, Minh-Hà P-D (2003). Statistical analysis of honeybee survival after chronic exposure to insecticides. Environ. Toxicol. Chem..

[62] EFSA. Towards an integrated environmental risk assessment of multiple stressors on bees: review of research projects in Europe, knowledge gaps and recommendations. EFSA J. 12, 3594 (2014).

[63] Courjaret R, Lapied B (2001). Complex intracellular messenger pathways regulate one type of neuronal alpha-bungarotoxin-resistant nicotinic acetylcholine receptors expressed in insect neurosecretory cells (dorsal unpaired median neurons). Mol. Pharmacol..

[64] Courjaret R, Grolleau F, Lapied B (2003). Two distinct calcium-sensitive and -insensitive PKC up- and down-regulate an alpha-bungarotoxin-resistant nAChR1 in insect neurosecretory cells (DUM neurons). Eur. J. Neurosci..

[65] Tan J, Galligan JJ, Hollingworth RM (2007). Agonist actions of neonicotinoids on nicotinic acetylcholine receptors expressed by cockroach neurons. Neurotoxicology.

[66] Thany SH, Courjaret R, Lapied B (2008). Effect of calcium on nicotine-induced current expressed by an atypical alpha-bungarotoxin-insensitive nAChR2. Neurosci. Lett..

[67] Bodereau-Dubois B (2012). Transmembrane potential polarization, calcium influx, and receptor conformational state modulate the sensitivity of the imidacloprid-insensitive neuronal insect nicotinic acetylcholine receptor to neonicotinoid insecticides. J. Pharmacol. Exp. Ther..

[68] Calas-List D, List O, Quinchard S, Thany SH (2013). Calcium pathways such as cAMP modulate clothianidin action through activation of α-bungarotoxin-sensitive and -insensitive nicotinic acetylcholine receptors. Neurotoxicology.

[69] Brunet JL, Badiou A, Belzunces LP (2005). *In vivo* metabolic fate of [14C]-acetamiprid in six biological compartments of the honeybee, Apis mellifera L. Pest Manag. Sci..

[70] Casida JE (2011). Neonicotinoid metabolism: compounds, substituents, pathways, enzymes, organisms, and relevance. J. Agric. Food Chem..

[71] Ford KA, Casida JE (2008). Comparative metabolism and pharmacokinetics of seven neonicotinoid insecticides in spinach. J. Agric. Food Chem..

[72] Simon-Delso N (2015). Systemic insecticides (neonicotinoids and fipronil): trends, uses, mode of action and metabolites. Environ. Sci. Pollut. Res. Int..

[73] Nauen R, Ebbinghaus-Kintscher U, Salgado VL, Kaussmann M (2003). Thiamethoxam is a neonicotinoid precursor converted to clothianidin in insects and plants. Pest Biochem. Physiol..

[74] Benzidane Y (2010). Effect of thiamethoxam on cockroach locomotor activity is associated with its metabolite clothianidin. Pest Manag. Sci..

[75] Lapied B, Le Corronc H, Hue B (1990). Sensitive nicotinic and mixed nicotinic-muscarinic receptors in insect neurosecretory cells. Brain Res..

[76] Thany SH (2011). Thiamethoxam, a poor agonist of nicotinic acetylcholine receptors expressed on isolated cell bodies, acts as a full agonist at cockroach cercal afferent/giant interneuron synapses. Neuropharmacology.

[77] Thany SH (2009). Agonist actions of clothianidin on synaptic and extrasynaptic nicotinic acetylcholine receptors expressed on cockroach sixth abdominal ganglion. Neurotoxicology.

[78] Ford KA, Casida JE (2006). Chloropyridinyl neonicotinoid insecticides: diverse molecular substituents contribute to facile metabolism in mice. Chem. Res. Toxicol..

[79] Karmakar R, Bhattacharya R, Kulshrestha G (2009). Comparative metabolite profiling of the insecticide thiamethoxam in plant and cell suspension culture of tomato. J. Agric. Food Chem..

[80] Iwasa T, Motoyama N, Ambrose JT, Roe RM (2004). Mechanism for the differential toxicity of neonicotinoid insecticides in the honey bee, Apis mellifera. Crop Prot..

[81] Sgolastra F (2020). Bees and pesticide regulation: Lessons from the neonicotinoid experience. Biol. Conserv..

